# An Integrated Care Platform System (C3-Cloud) for Care Planning, Decision Support, and Empowerment of Patients With Multimorbidity: Protocol for a Technology Trial

**DOI:** 10.2196/21994

**Published:** 2022-07-13

**Authors:** Malte von Tottleben, Katie Grinyer, Ali Arfa, Lamine Traore, Dolores Verdoy, Sarah N Lim Choi Keung, Igor Larranaga, Marie-Christine Jaulent, Esteban De Manuel Keenoy, Mikael Lilja, Marie Beach, Christopher Marguerie, Mustafa Yuksel, Gokce Banu Laleci Erturkmen, Gunnar O Klein, Pontus Lindman, Javier Mar, Dipak Kalra, C3-Cloud Research Team, Theodoros N Arvanitis

**Affiliations:** 1 empirica Gesellschaft für Kommunikations- und Technologieforschung mbH Bonn Germany; 2 Laboratoire d’Informatique Médicale et d’Ingénierie des Connaissances pour la e-Santé, LIMICS Inserm, Sorbonne Université, Université Paris 13 Paris France; 3 Kronikgune Institute for Health Services Research Barakaldo Spain; 4 Institute of Digital Healthcare (IDH), Warwick Manufacturing Group University of Warwick Coventry United Kingdom; 5 Basque Health Service (Osakidetza) Debagoiena Integrated Healthcare Organisation, Research Unit Arrasate-Mondragón, Guipúzcoa Spain; 6 Unit of Research, Education, and Development Östersund, Department of Public Health and Clinical Medicine Umeå University Umeå Sweden; 7 South Warwickshire University NHS Foundation Trust Warwick United Kingdom; 8 Software Research Development and Consultancy Cooperation, SRDC A.S. Ankara Turkey; 9 School of Business (Informatics) Örebro University Örebro Sweden; 10 Medixine Espoo Finland; 11 University of Gent Gent Belgium; 12 see Acknowledgements

**Keywords:** multimorbidity, polypharmacy, guidelines reconciliation, clinical decision support, personalized care plans, diabetes mellitus type 2, heart failure, depression, renal failure, acceptability, usability, evaluation, cost-benefit evaluation, predictive modeling

## Abstract

**Background:**

There is an increasing need to organize the care around the patient and not the disease, while considering the complex realities of multiple physical and psychosocial conditions, and polypharmacy. Integrated patient-centered care delivery platforms have been developed for both patients and clinicians. These platforms could provide a promising way to achieve a collaborative environment that improves the provision of integrated care for patients via enhanced information and communication technology solutions for semiautomated clinical decision support.

**Objective:**

The Collaborative Care and Cure Cloud project (C3-Cloud) has developed 2 collaborative computer platforms for patients and members of the multidisciplinary team (MDT) and deployed these in 3 different European settings. The objective of this study is to pilot test the platforms and evaluate their impact on patients with 2 or more chronic conditions (diabetes mellitus type 2, heart failure, kidney failure, depression), their informal caregivers, health care professionals, and, to some extent, health care systems.

**Methods:**

This paper describes the protocol for conducting an evaluation of user experience, acceptability, and usefulness of the platforms. For this, 2 “testing and evaluation” phases have been defined, involving multiple qualitative methods (focus groups and surveys) and advanced impact modeling (predictive modeling and cost-benefit analysis). Patients and health care professionals were identified and recruited from 3 partnering regions in Spain, Sweden, and the United Kingdom via electronic health record screening.

**Results:**

The technology trial in this 4-year funded project (2016-2020) concluded in April 2020. The pilot technology trial for evaluation phases 3 and 4 was launched in November 2019 and carried out until April 2020. Data collection for these phases is completed with promising results on platform acceptance and socioeconomic impact. We believe that the phased, iterative approach taken is useful as it involves relevant stakeholders at crucial stages in the platform development and allows for a sound user acceptance assessment of the final product.

**Conclusions:**

Patients with multiple chronic conditions often experience shortcomings in the care they receive. It is hoped that personalized care plan platforms for patients and collaboration platforms for members of MDTs can help tackle the specific challenges of clinical guideline reconciliation for patients with multimorbidity and improve the management of polypharmacy. The initial evaluative phases have indicated promising results of platform usability. Results of phases 3 and 4 were methodologically useful, yet limited due to the COVID-19 pandemic.

**Trial Registration:**

ClinicalTrials.gov NCT03834207; https://clinicaltrials.gov/ct2/show/NCT03834207

**International Registered Report Identifier (IRRID):**

RR1-10.2196/21994

## Introduction

Older age is associated with an increased accumulation of multiple chronic conditions called multimorbidity and includes functional and cognitive impairments. More than half of all older people have at least three chronic conditions and a significant proportion have 5 or more [1]. Chronic diseases take many forms such as hypertension, depression, diabetes mellitus type 2, and renal failure. They are the main reasons for poor health and a restricted activity. They impact over one-third of the European population and represent 70% of the health care expenditure in Europe [2].

The management of care for patients with multimorbidity is more complex and time consuming than those with a single disease [3]. Managing multiple diseases concurrently creates an added challenge for health service delivery and provision. Therefore, many individuals with chronic and long-term care needs experience shortcomings in the care they receive. One reason for this is the inconsistency across single-disease clinical guidelines when they cover situations with more than 1 disease. Current European medical models are often dictated by national clinical guidelines, which focus primarily on managing a single disease. Evidently, this can cause inconsistencies and provide contradictory information when providers are following more than 1 guideline for their patient. Furthermore, it can result in avoidable inefficiency for patients and health systems, for example, incompatible treatment regimens and duplicate clinical visits and tests [4].

Polypharmacy, induced by multimorbidity, is itself an important factor that leads to an increased risk of further complications in the provision of safe and effective care for patients, as well as the increased potential for adverse drug interactions and events [5]. Because the polypharmacy redundancy and duplication of medication are common, it not rare for elderly patients to be taking 9 or more medications concurrently [6]. This current approach of managing multimorbidity also fails to integrate care across providers and the interactions of chronic diseases and their treatments are overlooked [7]. As the number and complexity of health conditions increase with age, the type and number of care providers also increase. This often leads to fragmented care: it becomes significantly more difficult for providers to align and coordinate care teams and settings. This is exacerbated by poor interprofessional communication and lack of appropriate information-sharing infrastructure that exists in many health systems and even at local level. Without secure information exchange among the actors involved in health, social, and informal care services, it becomes almost impossible to reconcile potentially conflicting treatment plans or avoid potentially harmful interventions. An insufficient information exchange complicates the application of data-processing techniques developed under paradigms such as data science, machine learning, and artificial intelligence that could support medical decision making with information analysis and predictive models.

Moreover, patients and their informal caregivers often do not have a voice in the management of their own care. This can lead to patients feeling disempowered, less well informed, and therefore less likely to follow the treatment regime “imposed” on them. Among elderly people, noncompliance has a prevalence of 25%-75% and the likelihood rises in proportion to the number of drugs and daily doses prescribed [8]. There is an increasing need to focus care organization on a patient with multiple diseases, rather than targeting each disease separately. This requires a patient-centered approach: considering each patient’s multiple physical conditions, psychosocial conditions, and the realities of multimorbidity and polypharmacy. An interactive collaborative environment is needed to address these issues in the current care of patients with multimorbidities.

In response, C3-Cloud, a European Commission–supported Horizon 2020 innovation project, was created to pilot test collaborative computer platforms for patients and members of the multidisciplinary teams (MDTs) in 3 different European settings. The aims of the platform are to improve the provision of integrated care for patients with multimorbidity, resolve guideline conflicts (by reconciliation of varying, and potentially conflicting, recommendations from single-disease clinical guidelines), support clinical decision making through clinical decision support services, and facilitate communication among MDT members and with the patients through an interoperable platform (Multimedia Appendix 1). Traditional, “paper-based” health records have strong limitations for the integration of care or collaborative decision making and electronic health records (EHRs) attempt to widen the scope of health records [9]. As the health care landscape is ever changing, EHRs have the potential to replace paper records and add many more capabilities, beyond mere replication of data in an electronic format. New tools such as C3-Cloud can enhance the interaction among MDTs, patients, and their informal caregivers. The objective of this study is to determine the impact the platform will have on patients, MDT members, and health systems with the guiding research question being “Is the use of a personalized ICT tool that facilitates coordinated care planning, treatment optimization, and patient self-management acceptable to patients with multiple long-term conditions and their team of health professionals?”. The overall C3-Cloud system architecture is shown in Multimedia Appendix 2 and Multimedia Appendix 3 describes the main components of the C3-Cloud system.

The purpose of this paper is to present the research protocol of the C3-Cloud technology trial as a sustainable protocol guiding the development, testing, and evaluation of other interactive health care platforms targeting patients and MDT members.

## Methods

### Ethical Considerations

The study received favourable ethical approval from the three pilot regions: In the UK, the North of Scotland Research Ethics Committee approved the study (Integrated Research Application System (IRAS) project ID 224635, 25 May 2018); in Spain, the Basque Ethical Board - Comité de Ética de la Investigación con medicamentos de Euskadi (CEIm-E) - approved the study (PI2018006, 14 May 2018); in Sweden, the Northern Ethical Review Board - Regionala etikprövningsnämnden i Umeå – approved the study (Dnr 2018-3-31M, 5 April 2018). All associated amendments were also approved.

### Study Design

**Figure 1:**
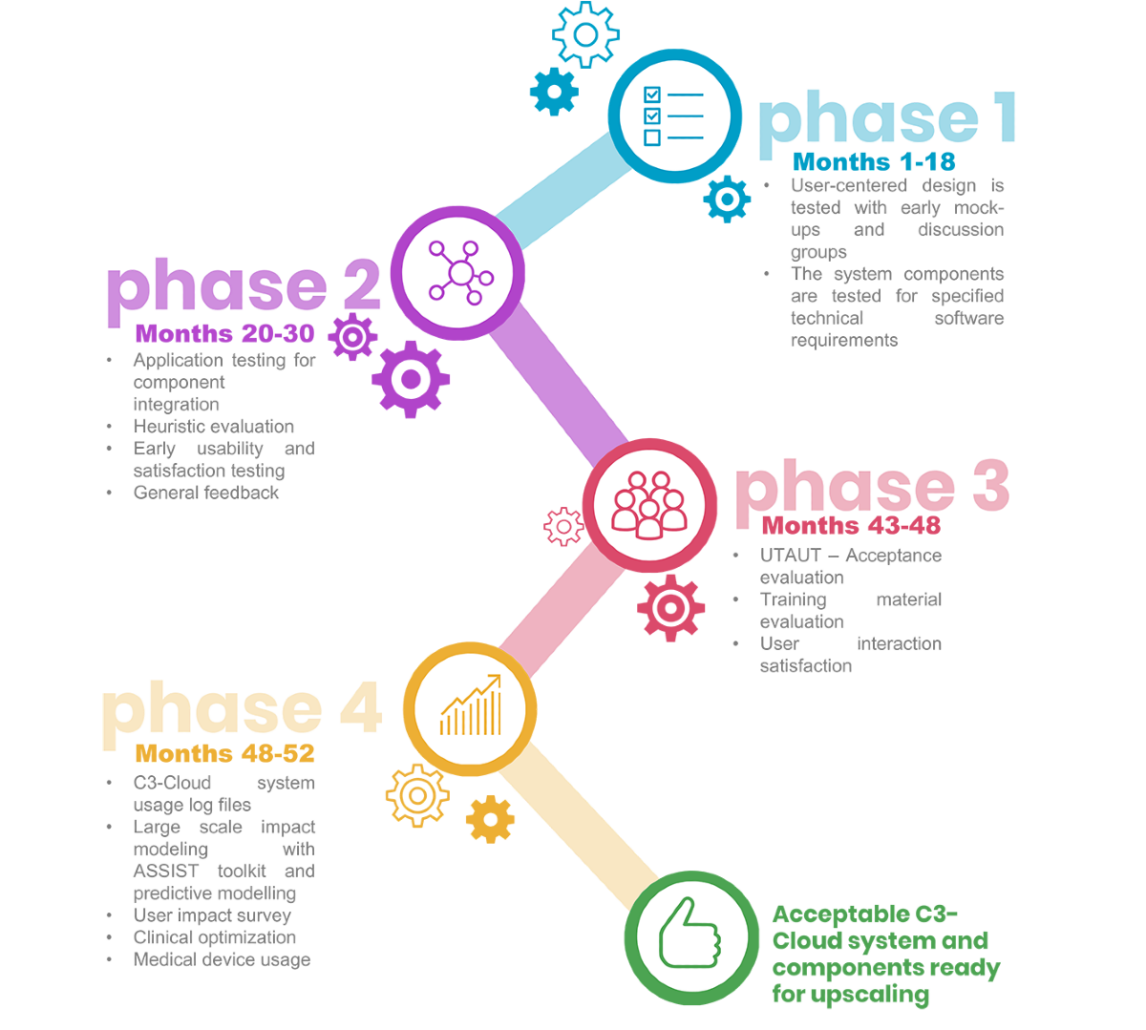
C3-Cloud system evaluation phases. UTAUT: Unified Theory of Acceptance and Use of Technology.

The C3-Cloud study used a mixed method research design to gain insights into the usability, acceptance, and usefulness of the C3-Cloud system. The project has developed an innovative care planning system called “C3-Cloud,” which was tested with patients, their informal caregivers, and health care professionals in the United Kingdom (South Warwickshire), Sweden (Region Jämtland Härjedalen), and Spain (Basque Country). The tests and evaluation activities generated data to assess the usability and usefulness of the C3-Cloud system as well as its acceptance and satisfaction among user groups. The study was designed to go through 4 evaluation phases. The adoption of phases corresponded to the study’s aims to develop the C3-Cloud system together with its users in an iterative approach of testing, feedback, and subsequent improvements, which is in line with the UK’s Medical Research Council (MRC) recommendations for carrying out complex interventions. The MRC suggests employing modeling and exploratory trials before aiming to carry out randomized controlled trials [10]. Following this advice, the project was designed to evaluate the system through 4 phases (Figure 1). All 4 phases are methodologically important for the successful testing of the C3-Cloud system. For this work, however, we focus on phases 3 and 4. The first 2 phases of the project will respectively be published in a separate paper. The user-centered design of phase 1 has been published deliberately already [11].

### Study Development and Timeline

After the initial evaluations and trials, the C3-Cloud system has been deployed at the pilot sites, which was followed by pilot phase 3 with a larger number of users compared with phases 1 and 2. The project aimed for a 6-month exploratory technology trial (phase 3), followed by a few months for system acceptance analysis and impact assessment of C3-Cloud in phase 4 (Figure 1). All test participants’ data as well as patient and clinician data were retrieved anonymously or anonymized and aggregated in the pilot sites before sharing the data sets for analysis. Control group data for the period of phase 4 were extracted from care centers in the pilot sites in April 2020. The anonymously retrieved information was on health care resource consumption. To ensure that data cannot be traced, the data extracts did not include demographic descriptors and identifiers. Data entry of resource utilization dates was manipulated automatically and randomly within a range of ±30 days for each entry.

### Study Setting

A technology trial was used to test the C3-Cloud system with MDT members, patients, and their informal caregivers (when available). The technology trial ran for 6 months and took place in 3 European pilot site regions: Basque Country, Spain; Jämtland Härjedalen, Sweden; and South Warwickshire, United Kingdom. Study settings included various locations that are relevant for the provision of health care, for example, health care centers, general practitioner offices, hospitals, and patients’ homes. The technology trial is registered at ClinicalTrials.gov (identifier: NCT03834207).

### Sampling and Recruitment

The recruitment period for patients started 3 months before the launch of the pilot test to allow sufficient time for the identification of eligible participants and obtaining informed consents, while also keeping the period between recruitment and piloting start as short as possible.

*MDT* members were contacted individually by pilot site managers using convenience sampling, considering their individual profiles, willingness to participate, and a few general inclusion criteria (Multimedia Appendix 4). This nonprobabilistic sampling involves the sampling of MDT members that are nearby, aiming for a total sample size of 62 across the 3 pilot sites.

For the iterative evaluation phase 3 and phase 4, we defined the patient number that we need to observe based on power calculations as the “observation goal,” which was 420 patients. An unknown number of patients may withdraw from their participation during the technology trial. Thus, we added a 25% dropout margin to the observation goal, summing up to 526 patients to be recruited for the piloting trial participation (ie, the “recruitment goal”). This dropout margin had been added because the average dropout rate across all clinical trials was expected at around 30% [12]. This, however, varies based on the participants level of income, education, etc. (the higher the income, education, etc., the lower the dropout rate) and because our pilot sites are located in higher-income areas with a comparatively higher level of education, it was concluded that the dropout rate would be slightly lower than the average. Furthermore, there are meta-analytical studies that similarly concluded with approximately 30% dropout rate, although with a wide variability [13]. This conclusion was further supported by the fact that many eligible patients already knew their MDTs; hence, a slightly higher-than-average cooperation and lower dropout rate were expected.

It was anticipated that several patients that were approached for participation would decline from the outset. Accordingly, the number of patients that were approached for participation (ie, the “approaching goal”) was 16% larger than the recruitment goal, summing up to 610 intervention patients across the 3 pilot sites. The number of comparator patients whose resource consumption data were monitored anonymously matched the intervention patient numbers at each pilot site.

Potential candidates were selected through each pilot site screening their databases for eligible patients who met the inclusion and exclusion criteria (Multimedia Appendices 4 and 5). No inclusion criteria for informal caregivers have been defined; however, exclusion criteria will be applied (Multimedia Appendix 4). Once the pilot sites have provided a list of eligible patients, they were randomized as study candidates to avoid selection bias. A first randomization round generated candidate lists that were 16% larger than the actual patient recruitment target per pilot site (including a 25% dropout rate), to adjust for patients that were approached but denied their participation.

Table 1 details the number of involved participants per pilot site and evaluation phase. The number of trial participants in each pilot site for evaluation layer 4 reads as follows: “minimum number of trial participants as calculated for the observation goal + 25% dropout rate (recruitment goal) + 16% denial rate (approaching goal)” and sums the total number of trial participants that were approached for participation.

**Table 1:** Number of trial participants to approach.

Pilot region	Phase 3: Exploratory trial for application evaluation			Phase 4: Monitoring to model large-scale impact			
	Patients	MDT^a^ members		Patients	Comparator patients	MDT members	
South Warwickshire	50	16		70 + 18 + 14	102	16	
Basque Country	50	16		175 + 44 + 35	254	16	
Jämtland Härjedalen	50	30		175 + 44 + 35	254	30	
Total	150	62		610	610	62	

^a^MDT: multidisciplinary team.

Research assistants at each site contacted (email, mail, phone, or face-to-face meetings) the selected study candidates and provided material and information about the study and its objectives. Supportive activities such as videos and presentations were sometimes used in a supplementary role to clarify any questions. Candidates who agreed to participate in the study had to sign an informed consent form for documentation to confirm they have read and understood the information and wanted to participate in the technology trial.

### Study Procedure

Early in the pilot technology trial a training was offered for all participants on how to use the platforms. The pilot technology trial was used to evaluate the user experience, satisfaction, and acceptability of the C3-Cloud application as well as the patient training material (phase 3). It also served to obtain anonymous patient data on resource usage for impact modeling and sustainability planning for upscaling C3-Cloud in phase 4. At the start of the trial, the patients had a care plan created on the C3-Cloud system that they developed and managed with their health care professionals during the study. Once the patient’s care plan was prepared, they were given access to the C3-Cloud system to view and update their care plan whenever they wished. Moreover, patients were able to send messages to their care team members via the system. The patients care plan in the C3-Cloud system was reviewed and adapted each time they visited a health care professional who was also taking part in the technology trial.

In the final phase of the project a comparison was made on the care and treatment received by patients that have used the system and those that have not (the comparator patient group). The comparator group data were taken from similar patients and retrieved anonymously from the local health care systems. Initial screening showed that a sufficient number of similar patients was available for data retrieval in the systems. These data contributed to determining the full impact of C3-Cloud by assessing the use of health care resources and medication prescription across both groups of patients (phase 4).

### Phase 3

#### Overview

This phase evaluated the user experience, satisfaction, and acceptance of the C3-Cloud application and patient training material by collecting evaluation data. Data were collected from a subset of participants (150 patients and 52 MDT members) from questionnaires they completed. Data on user experience and satisfaction were collected from the training material questionnaire and the validated Questionnaire on User Interaction Satisfaction (QUIS). The data collected on acceptability of the technology were obtained through a refined version of the validated Unified Theory of Acceptance and Use of Technology (UTAUT) questionnaire. The questionnaires were administered as an online survey a few weeks after the trial start and at the trial end. Trial participants were able to obtain the link to the online survey via the messaging service of the platforms, which ensured that no participant was contacted by the evaluation team directly, thereby avoiding confidentiality breaches. No incentives were provided for completing the surveys. The surveys were open for 3 weeks.

#### The Training Material Questionnaire

The training material was assessed from the survey that patients answered after the training period to determine whether they and their informal caregivers found the materials useful and informative. Data were gathered on user experience and whether users felt more knowledgeable about their conditions and if they felt enabled to use C3-Cloud to take care of their conditions after the training. It also considered whether the materials are a contributing factor to improve care coordination.

#### The Questionnaire for User Interaction Satisfaction

Similar to the early usability testing with a limited number of test users, the QUIS7 questionnaire [14] was used for both MDT members and patients when the technology trial was in full scope and it was administered partly after the initial user training at the beginning of the trial and partly at the end of the trial. The results from both questionnaires were compared and used, in an iterative fashion, for shaping the design and re-design of the C3-Cloud platform and for providing recommendations for areas of improvement.

#### The Unified Theory of Acceptance and Use of Technology Questionnaire

A C3-Cloud–adapted version of the UTAUT questionnaire [15] was used, covering some of the original UTAUT modules. The UTAUT is developed to predict individual adoption and use of new information technologies (ITs). It posits that individuals’ behavioral intention to use an IT is determined by 2 beliefs: *perceived usefulness*, which is defined as the extent to which a person believes that using an IT will enhance his or her job performance, and *perceived ease of use*, which is defined as the degree to which a person believes that using an IT will be free of effort. A shortened version of UTAUT was administered at the beginning of the pilot trial, just after participants have had training sessions on how to use the C3-Cloud components. This version included the following UTAUT modules: performance expectancy, effort expectancy, social influence, technology anxiety, adoption timeline, and behavioral intention. The questionnaire was administered again in a more comprehensive manner shortly before the end of the trial. This second version included the additional modules *cultural trends* and *language factors*. The results from the initial UTAUT were compared with the closure UTAUT questionnaire to evaluate the differences in acceptance and use of C3-Cloud technology over the trial duration.

### Phase 4

#### Overview

In Phase 4 modeling for large-scale impact of C3-cloud implementation after the technology trial was performed. The health and economic benefits of the intervention at the population level were evaluated to gain insights into savings that C3-Cloud could generate systemically in the long term. The digitalization of clinical patient histories and the coding of all contacts between patients and their MDTs into the EHR allowed to better understand the health demand of a population and to quantify the health and social burden of the disease. For this quantification, health care resource usage data of all patients were used and compared using modeling techniques with anonymous comparator patient data. The modeling tool used for this analysis has been developed by merging discrete event simulation modeling methods with a cost-benefit assessment tool [16]. The merger tool (Textbox 1) helped predict the return on investment and time to break even for integrated care implementation at a large scale. It was used to inform decision making in the management of integrated care in general and on the expected impact of scaling up the use of C3-Cloud. The aim was to develop a combined tool taking advantage of 2 existing approaches (ASSIST or Assessment and evaluation tools for telemedicine and telehealth [16] and predictive modeling [17,18]) that have been previously applied in other European projects such as CareWell [19] and SmartCare [20]. Merging them aimed to improve reliability and validity of the tool by incorporating the comprehensive perspective applied by ASSIST and the flexible engine developed in predictive modeling to represent mathematically the natural history of the disease. The conceptual model included not only the health system but also the complete set of stakeholders. Model parameterization was a challenge as data required for all stakeholders could not be obtained from evidence-based sources. The data focused on health care resource utilization, frequency of use of C3-Cloud components, and service satisfaction. The data needed for this type of modeling were taken from administering additional questionnaires to participants: the eCare Client Impact Survey (eCCIS) for patients; the eCare User Impact Survey (eCUIS) for MDT members; and a few additional questionnaire items for patients, MDT members, and informal care givers.

In addition, C3-Cloud log files and EHR exports were taken from EHRs of the intervention and comparator patients to evaluate the differences in health care resource utilization during the trial. This included, for example, changes to drug use, readmissions, number of adverse drug events, number of virtual sessions, or resource redistribution. The comparator group was taken from another practice and statistically adjusted for the differences based on historic data.

**Predictive modeling.**

**ASSIST Cost-Benefit Analysis Tool**

ASSIST (Assessment and evaluation tools for telemedicine and telehealth) is an assessment and evaluation tool originally developed for use in the context of telemedicine and telehealth services, specifically to assess the economic viability of telemedicine pilot projects [16]. During the validation phase, ASSIST was successfully applied by 5 telemedicine projects. A core aim of ASSIST is to facilitate the transposition of a pilot project into routine service operation and to support service providers in achieving a sustainable economic model where service benefits are higher than service costs. It also facilitates the transposition of a pilot project into routine service operation and supports service providers in achieving a sustainable economic model. The assessment process of the tool includes 3 steps:

1. *Service assessment model setup:* the service change is analyzed to identify the key components such as applicable governance and the reimbursement model, stakeholders, and financial impacts (costs and benefits on the stakeholders).

2. Data collection and monetization.

3. *Calculation of performance measure:* the main outcome measure is based on the ratio of total costs to total benefits, that is, including financial costs and benefits, resource costs and benefits, and intangible costs and benefits.

**Predictive Modeling**

Predictive modeling serves to calculate the budget impact analysis by reproducing the natural history of patients with multimorbidity in both the standard scenario and the new scenario related to the new intervention, which results in implementation, effectiveness (ie, how does the new intervention affect the number of contacts to health professionals), and costs. A budget impact analysis projects the burden of the target population within the conventional or baseline scenario and analyses how this burden would change if the intervention achieved the organizationally defined goal. The list of parameters for the modeling is listed in Multimedia Appendix 6.

#### The eCare Client Impact Survey and the eCare User Impact Survey

The eCCIS and eCUIS were used to evaluate the utility that the C3-Cloud application brought to the patients and MDT members. It measures how patients and informal carers perceived the utility of C3-Cloud. To this could be added scales addressing time use, willingness-to-pay, and perception of care integration. In addition, the overall satisfaction with the C3-Cloud system as a service, whether the service is worth the effort involved in using it, and whether the respondents would want to continue using the service or to use it again are evaluated.

#### Additional Questionnaire Items

A few questionnaire items have been added to the surveys to evaluate the impact of C3-Cloud implementation on patients, their informal caregivers, MDT members, and the wider service system. This was administered early in the trial and again at trial closure. The evaluation used open and closed questionnaires, targeting patients and MDT members. It evaluated the impact of the different software components and focused on the following evaluation topics: usefulness, ease of use/usability, safety, process quality and changes, and the respondents’ perspective on clinical optimization.

#### Medical Devices Questionnaire

In addition, medical sensor device usage and connected device usage were evaluated with a subset of patients at the Region Jämtland Härjedalen pilot site only. Patients were individually selected from the group of intervention patients at the discretion of local clinicians. The testing served to evaluate the technical possibility of including sensor and connected devices as part of the patient care planning.

## Results

**Figure 2:**
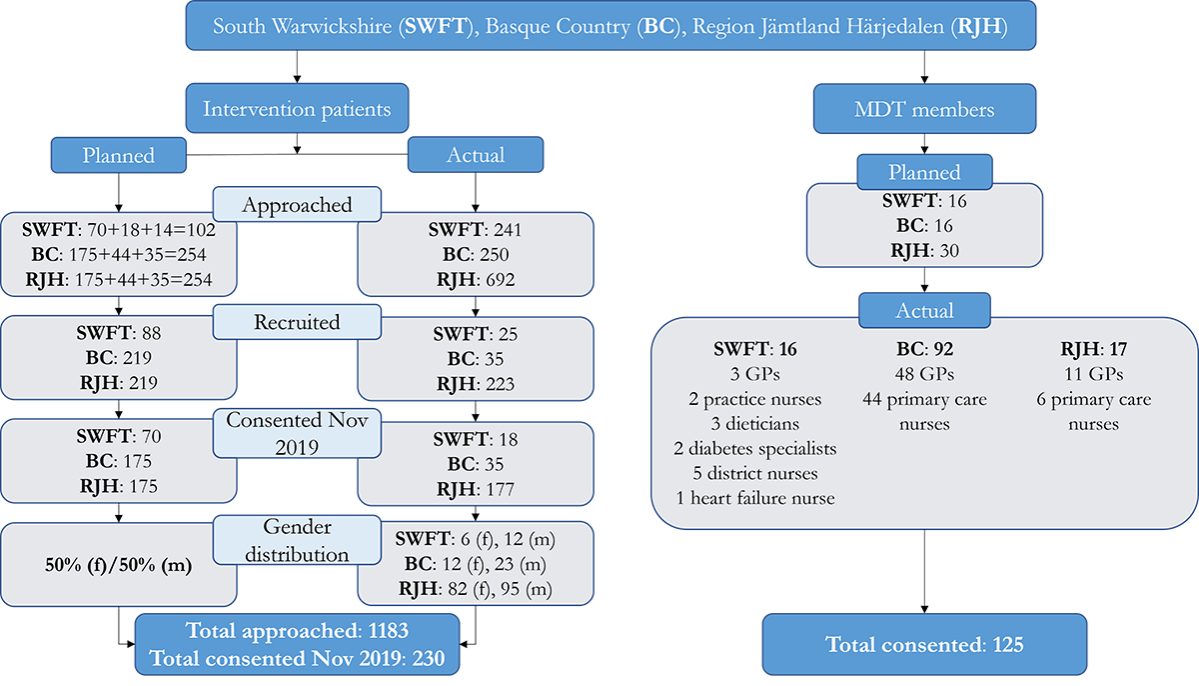
Number of trial participants (summary). GP: general practitioner; MDT: multidisciplinary team.

The pilot testing (phase 3) was carried out from November 2019 to April 2020. Difficulties were experienced in recruiting the envisaged number of trial participants, specifically with the intervention patients (Figure 2).

The technology trial protocol was submitted in several revisions to the 3 regional ethics committees in reflection of updates regarding project information that would be communicated to trial participants, the methods of accessing and using control group patient data, the recruitment procedure of patients, trial participants training, or adaptations to some questionnaires.

Data collection was completed in April 2020 with a total of 230 patients and 125 MDT members. The evaluation results of the pilot technology trial were analyzed and reported in a project report in June 2020 [21]. The main challenge with the technology trial evaluation was a limited data basis due to recruitment issues, a trial duration that was too short to show many significant differences in resource use for mild and moderate conditions that were included, and only a small number of returned surveys, mainly due to the COVID-19 pandemic, which dramatically reduced the use of the C3-Cloud platforms, and partially as the surveys were too lengthy. Although the methodological evaluation setup has proven feasible and useful, the evaluation results have limited validity and reliability.

Generally, acceptance and perceived usefulness of the C3-Cloud platforms are just slightly positive with only little variation between patients, informal care givers, and the MDT members.

A follow-up trial confirming the acceptance and evaluation of clinical impacts is also highly recommended.

Strengths of the approach taken are that it allowed for an early feedback to the software developers for further improvement of the software before starting the technology trial with an increased number of patients and MDT members in real settings. The combination of structured and unstructured feedback from the test sessions complemented each other. However, the validity of test results may be reduced based on the dependency on test participant’s fluency in the English language and the unequal distribution of test users across the pilot sites. Trial duration, patient and MDT member numbers, and usage frequency during the trial should be carefully considered.

## Discussion

This paper has presented the research protocol of the C3-Cloud technology trial as well as the development of the C3-Cloud platforms C3DP (Coordinated Care and Cure Delivery Platform) and PEP (Patient Empowerment Platform). C3-Cloud has developed a modified impact modeling tool in phase 4 (a merger of the ASSIST tool and predictive modeling) for informing integrated care management on a large-scale deployment potential of systems such as C3DP and PEP. The full results were reported in a project report [21]. The number of patients and MDT members varies across the 3 pilot sites based on convenience sampling as participation depends fully on the commitment of the pilot site organizations. Socioeconomically, it was determined that general practitioner and nurse consultations (0.63 and 0.74 times less likely), nurse home visits (0.45 times less likely), and the use of accident and emergency services (0.57 times less likely) have developed positively for patients using C3-Cloud, while nurse telephone consultations increased (1.6 times more likely). An overall positive systemic socioeconomic return of 228% (Basque Country, Spain), 285% (South Warwickshire, United Kingdom), and 399% (Jämtland Härjedalen, Sweden) was modeled for the 3 pilot sites [21].

The C3-Cloud system is designed in such a way that patients can work more closely with their health care professionals to create, develop, and manage their personal care plans. The platforms enable care plans to be personalized for multimorbid conditions through systematic and semiautomatic reconciliation of digitally represented clinical guidelines. Their commitment was crucial to conduct the technology trial throughout the different phases.

The research design leans on the MRC guidance for complex interventions [10]. The usefulness of complex interventions is determined also by the way they are implemented [22]. At the time of running the evaluation, C3-Cloud was in an early development and implementation phase and solutions needed thorough testing along various dimensions to better understand the benefits of information and communication technology in health care and to respond to the challenge of implementing complex interventions.

The results are available as open published results and to some extent as open-source software for other parties to make use of. Parts of the present technical solutions will be used in the follow-up project AdLife [23] or may be offered for routine expanded use in the 3 pilot regions and of course also in a wider scale throughout these countries, using the spin-off entity “C3-Cloud Partnership Ltd.”

The strong inclusion and commitment by the public health care organizations in the 3 regions imply that there is a strong probability of the results to be transformed into routine improved health care services for this important group of patients. Future research may include the possible reorganization of multiprofessional care services for elderly patients using collaboration tools such as C3-Cloud as well as by establishment of more decision support tools based on clinical guidelines for other conditions than the 4 diseases tested in the project.

## Appendix

Multimedia Appendix 1 C3-Cloud platform aims.

Multimedia Appendix 2 Overall C3-Cloud architecture.

Multimedia Appendix 3 C3-Cloud terminology.

Multimedia Appendix 4 Inclusion and exclusion criteria for patients and informal caregivers.

Multimedia Appendix 5 Diagnosis codes used for patient screening.

Multimedia Appendix 6 Full set of parameters for the predictive modeling.

## Abbreviations

ASSIST: Assessment and evaluation tools for telemedicine and telehealth
eCCIS: eCare Client Impact Survey
eCUIS: eCare User Impact Survey
EHR: electronic health record
IT: information technology
MDT: multidisciplinary team
MRC: Medical Research Council
QUIS: Questionnaire on User Interaction Satisfaction
UTAUT: Unified Theory of Acceptance and Use of Technology
