# Supplementary figures and images for: An Integrated Care Platform System (C3-Cloud) for Care Planning, Decision Support, and Empowerment of Patients With Multimorbidity: Protocol for a Technology Trial
Source: JMIR Res Protoc. 2022 Jul 13;11(7):e21994. doi: 10.2196/21994 (PMC9330187; doi:10.2196/21994)

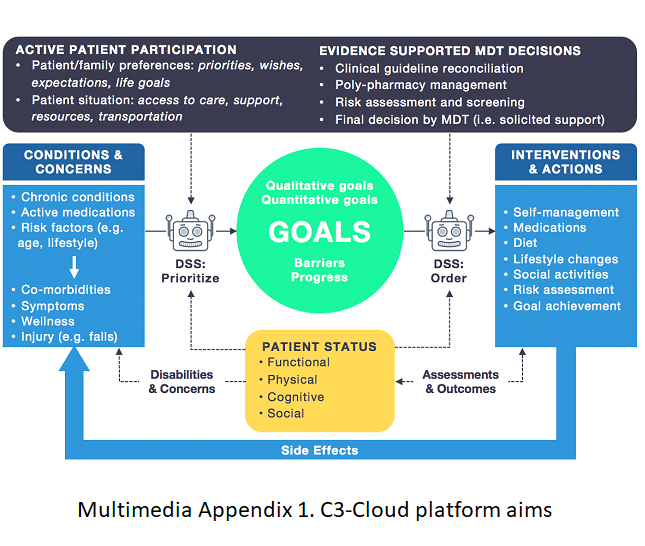

Supplement: Multimedia Appendix 1 [file resprot_v11i7e21994_app1.png]

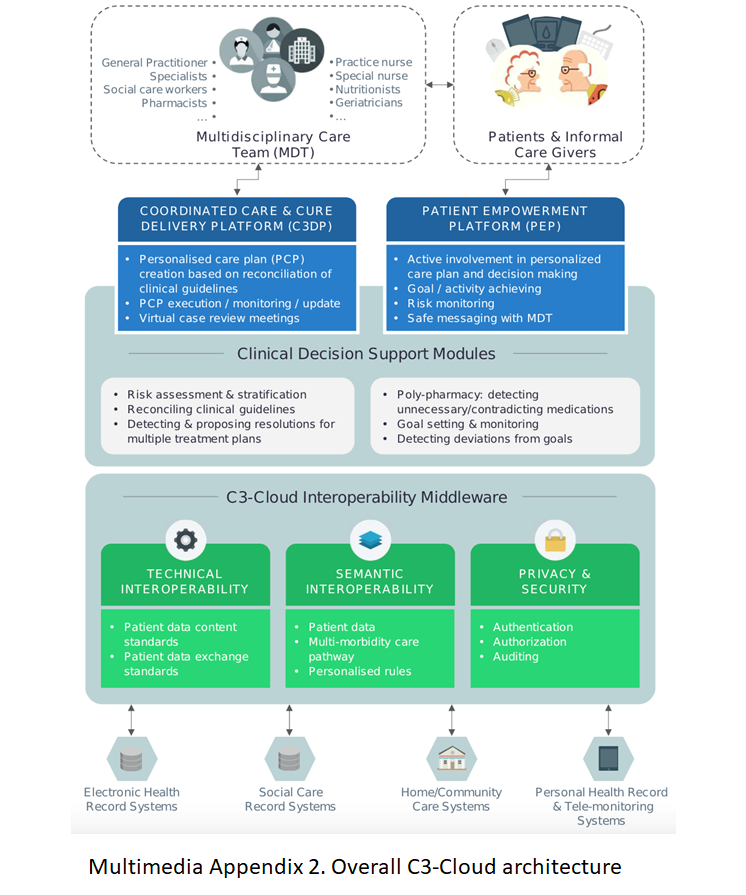

Supplement: Multimedia Appendix 2 [file resprot_v11i7e21994_app2.png]
