# Supplementary material for: An Integrated Care Platform System (C3-Cloud) for Care Planning, Decision Support, and Empowerment of Patients With Multimorbidity: Protocol for a Technology Trial
Source: JMIR Res Protoc. 2022 Jul 13;11(7):e21994. doi: 10.2196/21994 (PMC9330187; doi:10.2196/21994)
Supplement: Multimedia Appendix 3 [file resprot_v11i7e21994_app3.docx]

| *The C3-Cloud system* aims to facilitate the realization of two main components: the Coordinated Care & Cure Delivery Platform (C3DP) and the Patient Empowerment Platform (PEP). It also involves a variety of other components: The clinical decision support modules (CDSM), the interoperability middleware which includes modules of technical and semantic interoperability, as well as privacy and security. All these components constitute the solution that will be used for the technological trial of the C3-Cloud application. |
| --- |
| *The Coordinated Care and Cure Delivery Platform (C3DP) is* an innovative online means for Multi-Disciplinary Team (MDT) members to collaboratively manage (execute, monitor, update) the integrated personalized care plans for patients with multi-morbid conditions. The health professional will have a personal log-in account. MDT members and patients have the ability to send messages via the messaging portal to each other. The aim of the C3DP is the creation and execution of personalised care plans for multi-morbid patients, with the help of Clinical Decision Support Modules (CDSM) for recommendation reconciliation, poly-pharmacy management and goal setting. |
| *The Patient Empowerment Platform (PEP)* is for patients and their informal caregivers to access their care plans online and support them in self-managing their care. It aims to improve the interaction between patients and health professionals and to collect relevant information (home-based self-measurement data on blood pressure and weight) to enable the monitoring of care plan related activity status and progress. Clinicians can send medication or lifestyle change reminders as well as answer questions patients might have about their care. |
| *The Technical Interoperability Suite (TIS)* enables health data sharing between C3-Cloud high-level components, including information systems of local care providers and tele-monitoring devices, in order to support integrated care plan development, care plan progress monitoring and evaluation, as well as patient engagement across multiple care settings. |
| *The Semantic Interoperability Suite (SIS)* handles structural mappings among different information models and resolves semantic mismatches due to use of different terminology systems and different compositional aggregations to represent the same clinical concept. |
| *The Security and Privacy Suite (SPS)* guarantees authentication and authorisation of members of the MDT while they are managing personalised care plans of patients and accessing sensitive personal data. The SPS ensures that data exchange within and across C3-Cloud software components is encrypted and audited properly. |
| *Clinical Decision Support Module (CDSM)* provides guideline-based alerts, reminders or suggestions to support clinical pathways, and implements widely accepted polypharmacy criteria and risk assessment algorithms to support care plan reconciliation and patient risk stratification. |

**Multimedia Appendix 3**. C3-Cloud terminology.
