# Supplementary material for: An Integrated Care Platform System (C3-Cloud) for Care Planning, Decision Support, and Empowerment of Patients With Multimorbidity: Protocol for a Technology Trial
Source: JMIR Res Protoc. 2022 Jul 13;11(7):e21994. doi: 10.2196/21994 (PMC9330187; doi:10.2196/21994)
Supplement: Multimedia Appendix 4 [file resprot_v11i7e21994_app4.docx]

| Inclusion and exclusion criteria | |
| --- | --- |
| Inclusion criteria patients | Patients must be aged 55 or older  They are multimorbid patients that suffer from two or more of the following four conditions in various disease combinations:   - Type II Diabetes - Renal Failure with estimated or measured glomerular filtration rate (GFR/eGFR) of 30 – 59 - Heart Failure in compliance with NYHA I-II (New York Heart Association classification of heart failure) - Mild or moderate Depression   They, or their informal caregiver, have stable access to the internet and at least one of the following devices readily available to use the C3-Cloud components: Computer; Notebook; Smartphone; Tablet. This includes the use of Internet Browsers to open the C3-Cloud patient dashboards online. |
| Exclusion criteria patients | They suffer from any of the following conditions:   - Severe Renal Failure with GFR/eGFR <30 - Severe Heart Failure in compliance with NYHA III-IV - Severe Depression   They live in a care institution, for instance in a residential home or nursing home.  They or their informal caregivers do not pass the ICT Handling Self-Check (i.e. they do not have access to suitable IT devices and do not have some familiarity with the use of ICT).  They are unable to give informed consent.  They have disabilities or other health conditions that would prevent their active involvement in the study project or which prevent them from carrying out essential functions of the trial.  They have other debilitating conditions that impair their decision making capability or their life expectancy (e.g. end-of-life patients or cancer patients). Patients with further chronic diseases and other co-morbidities or symptoms, for example, frailty, sleeping problems, malnourishment or anxiety, will not be excluded from recruitment. Informal caregivers who pass the ICT Handling Self-Check can substitute for the patient if the patient does not pass the ICT Handling Self-Check – the patient-informal caregiver pair can still be recruited.  They do not speak the regional language: English for SWFT; Spanish for the Basque country; Swedish for RJH.  Their health care expenses are covered by a private insurance: in the C3-Cloud pilot sites, private insurances have no data exchange with EHRs. |
| Inclusion criteria for MDT members | The MDT member would normally be involved in the selected patients care.  The MDT member should be open to new ways of working, specifically as part of a MDT (including GPs; consultant nurses or specialist nurses; district nurses; social workers; consultants; physiotherapists or pharmacists).  The MDT member should be open to the use of new technology: MDT members do not have to be technologically knowledgeable, but they should be willing to learn how to use technology to support their work.  They take care of patients with the four target conditions. |
| Exclusion criteria for informal caregivers | They are aged 17 or below.  They are not available most of the time in person or via telephone, email, SMS or other means to respond to calls for help.  They do not have some familiarity with the use of ICT or do not have the capability to help the patient out with ICT usage if necessary.  They are unable to provide informed consent for trial participation.  They do not have stable access to the internet and at least one of the following devices readily available to use the C3-Cloud components: Computer; Notebook; Smartphone; Tablet. This includes the use of Internet Browsers to open the C3-Cloud patient dashboards online.  They have debilitating conditions that impair their decision-making capability.  They do not speak the local language: English for SWFT; Spanish for the Basque country; Swedish for RJH |
