# Supplementary material for: An Integrated Care Platform System (C3-Cloud) for Care Planning, Decision Support, and Empowerment of Patients With Multimorbidity: Protocol for a Technology Trial
Source: JMIR Res Protoc. 2022 Jul 13;11(7):e21994. doi: 10.2196/21994 (PMC9330187; doi:10.2196/21994)
Supplement: Multimedia Appendix 5 [file resprot_v11i7e21994_app5.docx]

| Condition | READ Codes: South Warwickshire | ICD-10 / ICD-9 Basque Country and Jämtland Härjedalen |
| --- | --- | --- |
| Type II Diabetes | Type 2 Diabetes: C10F** (including all codes below in the code tree) | Diabetes = E11  Diabetes with CF= E11.2 or I13.0  Diabetes with complications = E11.8P  ATC = N06A |
| Renal Failure with eGFR/ GFR 30 – 59 (measured or estimated glomerular filtration rate) | K05* (Chronic renal failure) and all codes below in the tree.    1Z1* (chronic renal impairment) (including all codes below in the code tree) | RF = N18.9 or I12.0 or I13.1 or N19.9 or N19.-P or N18.2 to N18.5  Hypertension +RF+ CF = I13.2 |
| Heart Failure in compliance with NYHA I-II (New York Heart Association classification of heart failure) | C58* (including all codes below in the code tree)    420300004 (NYHA Class I) and 421704003 (NYHA Class II) classification will be an individual check by a GP | CF= I50 or I11.0  Diabetes with CF= E11.2 or I13.0  Hypertension +RF+ CF = I13.2 |
| Mild or moderate depression in adults | Anxiety with depression – include all. (Read Code: E2003)  Depression NOS – include all. (Read Code: Eu32z-1)  Depressive episode, unspecified – include all. (Read Code: Eu32z)  Endogenous depression – include all. (Read Code: E112-4)  Reactive depression NOS include all. (Read Code: Eu32z-4)  Chronic depression – include all. (Read Code: E2B1)  Recurrent depression – include all. (Read Code: E1137)  Endogenous depression – recurrent – include all. (Read Code: E113-1)  Low Mood – include all. (Read Code: 1BT-1) | Depression = F32.9   or F32.1  or F33.1  or F32.0  or F32-  or F33- |
