# Supplementary material for: An Integrated Care Platform System (C3-Cloud) for Care Planning, Decision Support, and Empowerment of Patients With Multimorbidity: Protocol for a Technology Trial
Source: JMIR Res Protoc. 2022 Jul 13;11(7):e21994. doi: 10.2196/21994 (PMC9330187; doi:10.2196/21994)
Supplement: Multimedia Appendix 6 [file resprot_v11i7e21994_app6.docx]

| Age range |
| --- |
| Sex |
| Diseases that patient has (ICD-10 codes) |
| Patient Diseases Identification Date |
| Patients Dropout status |
| Patient Dropout Date |
| All Contact Dates between patient and Primary Care Doctor at Centre, (+ - 30 days) |
| All Contact Dates between patient and Primary Care Doctor at Home , (+ - 30 days) |
| All Contact Dates between patient and Primary Care Doctor by Telephone, (+ - 30 days) |
| All Contact Dates between patient and Primary Care Nurse at Centre, (+ - 30 days) |
| All Contact Dates between patient and Primary Care Nurse at Home, (+ - 30 days) |
| All Contact Dates between patient and Primary Care Nurse by Telephone, (+ - 30 days) |
| All Contact Dates between patient and Secondary Care in Cardiology, (+ - 30 days) |
| All Contact Dates between patient and Secondary Care in Endocrinology, (+ - 30 days) |
| All Contact Dates between patient and Secondary Care in Nephrology, (+ - 30 days) |
| All Contact Dates between patient and Secondary Care in Psychiatry, (+ - 30 days) |
| All Contact Dates between patient and Secondary Care in Internal Medicine, (+ - 30 days) |
| All Contact Dates between patient and A&E Services, (+ - 30 days) |
| All Contact Dates between patient and Hospitalization and the number of days hospitalized, (+ - 30 days) |
| All Contact Dates between patient and Home Hospitalization, (+ - 30 days) |
| Unit costs per Contact with Primary Care Doctor at Centre |
| Unit costs per Contact with Primary Care Doctor at Home |
| Unit costs per Contact with Primary Care Doctor by Telephone |
| Unit costs per Contact with Primary Care Nurse at Centre |
| Unit costs per Contact with Primary Care Nurse at Home |
| Unit costs per Contact with Primary Care Nurse by Telephone |
| Unit costs per Contact with Secondary Care in Cardiology |
| Unit costs per Contact with Secondary Care in Endocrinology |
| Unit costs per Contact with Secondary Care in Nephrology |
| Unit costs per Contact with Secondary Care in Psychiatry |
| Unit costs per Contact with Secondary Care in Internal Medicine |
| Unit costs per Contact with A&E Services |
| Unit costs per Contact with Hospitalization or per Hospitalization Day |
| Unit costs per Contact with Home Hospitalization or per Home Hospitalization Day |
| Names of Drugs Prescribed to the patient |
| Unit Dose of Drugs Prescribed to the patient |
| Frequency of Drugs Prescribed to the patient |
| Days or Date of Drugs Prescribed to the patient |
| Cost per Unit Dose of Drugs Prescribed to the patient |

**Multimedia Appendix 6.** Full set of parameters for the predictive modeling.
